# A post traumatic capsulorhexis

**DOI:** 10.11604/pamj.2020.35.134.20705

**Published:** 2020-04-20

**Authors:** Kawtar Belkhadir, Ouafaa Cherkaoui

**Affiliations:** 1 Département d’Ophtalmologie Unit A, Hôpital des Spécialités, Faculté de Médicine et Pharmacie, Université Mohammed V, Rabat, Maroc

**Keywords:** Ocular trauma, post traumatic cataract, capsulorhexis

## Image in medicine

We report the case of a patient aged 50 years, with no pathological history, who consulted for a decrease in visual acuity secondary to an ocular trauma of the left eye occurred 6 months ago. The clinical examination found a visual acuity reduced to luminous perception, a clear cornea, an anterior chamber of good depth. Examination after pupillary dilation revealed an anterior capsule broken, in the manner of an incomplete circular anterior capsulorhexis, with retraction of the anterior capsule in superior temporal area, and yellowish pupillary reflection. The examination was completed by a B-mode ultrasonography which revealed a vitreous dislocated lens nucleus with vitreous organization in favor of intravitreal hemorrhage secondary to the trauma associated with an old retinal detachment. In view of the severity and the age of the post-traumatic lesions, therapeutic abstention was advocated. Ocular contusion trauma is a common cause of ophthalmic emergency consultation. An early and complete examination is necessary in order to carry out an exhaustive lesion assessment and to allow an early and adequate management in order to avoid the potentially blinding sequelae of these affections.

**Figure 1:**
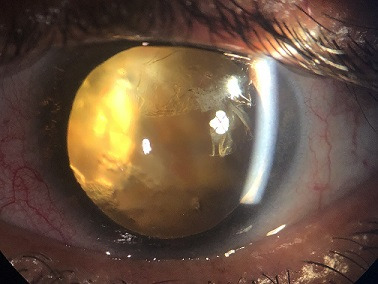
Image of the left eye showing a retraction of anterior capsule

